# The augmented clinician as a framework for human-AI collaboration in mental healthcare

**DOI:** 10.3389/fpsyt.2026.1729175

**Published:** 2026-03-02

**Authors:** Qian-Nan Ruan, Shuang-Qian Hu, Zhi-Hui ShangGuan, Sun-Meng Zhou

**Affiliations:** 1 Wenzhou Seventh People’s Hospital, Wenzhou, China; 2 Wenzhou Center for Disease Control and Prevention, Wenzhou, China; 3 The Affiliated Kangning Hospital of Wenzhou Medical University, Zhejiang Provincial Clinical Research Center for Mental Health, Wenzhou, China

**Keywords:** artificial intelligence, augmented clinician, ethics, human-AI collaboration, mental health

## Abstract

The global mental health system faces an unprecedented crisis of access, with demand for care far outstripping the supply of trained professionals. Artificial Intelligence (AI) has emerged with immense promise to bridge this gap through scalable and accessible solutions. However, its rapid and often unregulated deployment introduces significant ethical perils, including the dehumanization of care, the perpetuation of societal biases, and the risk of clinical harm. This perspective argues against the pursuit of autonomous AI therapists and instead advocates for the Augmented Clinician model. This framework positions AI as a sophisticated and transparent supportive tool that enhances, rather than replaces, human clinicians. By delegating data-intensive and administrative tasks to AI, clinicians can dedicate more time to the irreplaceable human elements of therapy such as empathy, nuanced judgment, and fostering the therapeutic alliance. We propose that this collaborative human-AI synergy is the most effective and ethically sound path to harness technology’s power while ensuring mental healthcare remains fundamentally human-centered.

## 1 Introduction

The 21st-century mental healthcare landscape presents a contrast: while public awareness has increased, the global care infrastructure faces significant systemic challenges (1). Estimates indicate that over one billion people live with a mental health disorder, yet a substantial treatment gap persists, leaving a majority of individuals without adequate support (2). This systemic deficiency, attributed to chronic underinvestment and workforce shortages, necessitates innovation in care delivery models.

Artificial Intelligence (AI) has entered this landscape as a promising solution, offering scalability, 24/7 availability, and cost-effectiveness that traditional models cannot match (3). The potential to deliver evidence-based support via smartphones represents a paradigm shift. This promise, however, is shadowed by significant risk. A burgeoning market of direct-to-consumer AI applications is rapidly filling the care vacuum, often with minimal clinical validation or regulatory oversight (4). This dynamic threatens to establish a de facto standard of care dictated by market forces rather than evidence-based principles.

The central question is not whether to use technology, but how to integrate it safely, effectively, and equitably. This article presents a perspective that the most viable path forward is not automation but augmentation. We argue for the Augmented Clinician model, a framework where AI functions as a subordinate partner to the human professional (5). This approach seeks to leverage AI’s computational strengths to re-humanize care, freeing clinicians to focus on the essential therapeutic relationship that remains the most reliable predictor of positive outcomes (6).

## 2 Current advances and emerging risks in mental health AI

The application of AI in mental healthcare is no longer theoretical; it is an active and rapidly evolving field. In diagnostics and prognostics, AI offers new levels of objectivity (7). Machine learning models can synthesize vast, heterogeneous datasets from neurophysiological signals, clinical notes, and even social media to identify subtle patterns of illness (8). A transformative innovation is digital phenotyping, which uses passive sensor data from personal smartphones to create an objective, real-world measure of a patient’s functioning (9). This can reveal behavioral changes that signal the onset of a manic or depressive episode, enabling proactive intervention. Predictive models show remarkable accuracy in identifying at-risk youth using simple questionnaire data, providing clinicians with a powerful tool for preventive care (10).

The most visible advance is the therapeutic chatbot. Platforms like Wysa and Woebot deliver structured interventions based on Cognitive Behavioral Therapy (CBT) and have demonstrated efficacy in reducing symptoms of depression and anxiety in multiple randomized controlled trials (11). Users often value the anonymity and constant availability of these tools. This has led to the phenomenon of users forming a “digital therapeutic bond” with chatbots, with research indicating that this bond can be comparable to that measured in human psychotherapy (12).

This finding, however, must be interpreted with caution. This bond is unidirectional, reflecting a profound human need for connection that can be projected onto a non-sentient algorithm. To conflate this simulated relationship with the co-constructed, reciprocal nature of a true human therapeutic alliance is a category error. Relying on this fragile bond fosters dependency on a system that cannot truly understand or challenge the user, creating significant long-term psychological risks. The recent emergence of powerful Large Language Models (LLMs) like GPT-4 has amplified this concern. Millions are turning to these highly conversational AIs for mental health support in a regulatory vacuum, creating an uncontrolled risks. Recent technical evaluations indicate that these models can systematically violate ethical standards in mental health practice, including inappropriate responses to crisis situations (13).

## 3 Preserving the human core amid algorithmic perils

Before integrating any technology, we must define what is non-negotiable. Decades of research confirm that the quality of the therapeutic alliance is the single most robust predictor of successful treatment outcomes across all modalities (14). This alliance is built on collaboration, trust, and a shared understanding of goals. It is a dynamic process co-constructed between two human beings.

The medium for this alliance is empathy. Human empathy is a complex process involving both affective empathy (the ability to feel with another) and cognitive empathy (the ability to understand another’s perspective) (15). AI, as a computational system, is fundamentally incapable of affective empathy. It has no biological substrate for emotion (16). At its best, AI can perform a sophisticated simulation of cognitive empathy, analyzing user input to generate a statistically appropriate response. Crucially, recent advancements in LLMs have made this simulation increasingly indistinguishable from human interaction to the lay observer. This high-fidelity mimicry heightens the risk of deception, as patients may feel deeply understood by a system that, in reality, understands nothing. This makes the genuine, shared interpersonal experience at the core of the therapeutic bond irreplaceable.

Beyond these relational limitations, AI models carry inherent technical risks. Many powerful deep learning models are “black boxes” whose internal decision-making is opaque even to their creators (17). In a clinical setting, this opacity is a critical failure. It erodes trust, prevents the debugging of errors, and creates a crisis of accountability when a harmful recommendation is made. A recommendation that cannot be understood and defended by a clinician is not clinically valid. Therefore, explainability is not a feature to be traded for accuracy; it is a prerequisite for safe deployment.

Perhaps the most insidious risk is algorithmic bias. AI models learn from data, and if that data reflects existing societal inequities, the AI will learn, reproduce, and scale those same biases under a veneer of technological objectivity (18). For instance, a widely used healthcare algorithm was found to systematically underestimate the health needs of Black patients because it used historical spending as a proxy for need, reflecting systemic inequities in access to care. In mental health, this risk is acute. Models trained on unrepresentative data or biased clinical records threaten to deepen health disparities for marginalized communities (19).

## 4 The augmented clinician model

Given that the human elements of therapy are irreplaceable and that autonomous AI carries profound risks, the clearest path forward is human-AI collaboration. We propose the Augmented Clinician model as a guiding philosophy. This framework reframes AI from a replacement to a powerful instrument that enhances the clinician’s cognitive and administrative capacities. The core principle is that by delegating appropriate tasks to the machine, the clinician is freed to focus on the uniquely human aspects of care.

This model is defined by four key tenets. First, the human-in-the-loop is a non-negotiable default. The human clinician must retain ultimate authority, accountability, and responsibility for all clinical decisions (20). Second is task delegation based on complementary strengths. Computationally intensive work like data analysis and pattern recognition is assigned to the AI, while relationship-intensive work requiring empathy and ethical deliberation is reserved for the human. Third, the tools must be transparent and explainable. Clinicians must be able to scrutinize the AI’s reasoning to evaluate its validity and identify potential bias. Fourth, all technology use must occur within the context of the co-constructed therapeutic alliance, with the patient’s full informed consent.

To illustrate, consider a patient presenting with depression. An AI decision support system could synthesize the patient’s electronic health record, lab results, and intake questionnaires. It would cross-reference this data with clinical literature to generate a concise, explainable brief for the clinician, highlighting potential comorbidities, risk factors, and evidence-based treatment options. The clinician, having reviewed this brief, can then devote the entire session to building rapport and exploring the patient’s subjective experience. However, for this workflow to be viable, it is imperative to address the risk of cognitive overload. The interface must be designed with user-centered principles to seamlessly integrate into clinical workflows, ensuring that verifying AI outputs does not become a burden that detracts from patient care. While AI functions as a “cognitive exoskeleton” that ensures comprehensive data integration, the final clinical formulation remains a product of human judgment and shared decision-making with the patient. This model uses AI not to automate care, but to re-humanize it (**Table 1**).

**Table 1:** Key ethical risks and mitigation strategies within the augmented clinician model.

Ethical principle and key risk	Proposed mitigation strategy
Transparency and informed consent Patients are unaware of AI’s role, leading to a loss of autonomy and trust	Mandate explicit disclosure of AI use in consent forms. Clinicians must explain the tool’s purpose, limitations, and the patient’s right to opt-out
Bias and equity AI models trained on unrepresentative data amplify health disparities for marginalized groups	Require fairness audits on AI tools. Use Explainable AI to identify biased reasoning. Mandate clinician oversight to contextualize AI output for individual patients
Human oversight and accountability Over-reliance on “black box” AI abrogates professional responsibility and makes it impossible to assign liability for errors	Legislate a “human-in-the-loop” requirement for all high-risk clinical decisions. Prohibit autonomous therapeutic AI. The clinician retains ultimate accountability
Privacy and security Sensitive mental health data is breached or used unethically for commercial purposes	Enforce strict compliance with data protection laws like HIPAA. Use privacy-preserving techniques. Mandate clear data use policies
Safety and efficacy Unvalidated AI tools provide harmful or inaccurate advice, particularly in crisis situations	Establish rigorous validation standards for AI medical devices (e.g., via FDA/MHRA). Prohibit AI use for autonomous crisis response. AI alerts must be routed to a human professional

## 5 Discussion and future directions

Translating the Augmented Clinician model from concept to practice requires a concerted, multi-stakeholder effort. The rapid pace of technological development is far outstripping the capacity of our regulatory and educational systems. A responsible transition depends on building robust governance, transforming professional training, and strategically redirecting research.

First, we need a coherent governance framework. This should be built on international ethical principles like those from the WHO and employ a risk-based approach to regulation, as mandated by the EU Artificial Intelligence Act (Regulation (EU) 2024/1689). Under this framework, AI systems used in healthcare are often classified as “high-risk,” requiring stringent validation and human oversight (Article 14) (21). National and state-level legislation, such as the Illinois Wellness and Oversight for Psychological Resources Act (Public Act 104-0054), which explicitly prohibits AI from making independent therapeutic decisions or engaging in “therapeutic communication” without licensed professional oversight, is crucial for codifying the human-in-the-loop principle into law (22).

Second, we must address the significant competency gap among healthcare professionals by embedding AI literacy into professional education. Medical, psychology, and nursing curricula must include foundational training on the principles, capabilities, and limitations of AI in clinical contexts (23). Crucially, this training must cultivate a mindset of healthy skepticism and critical appraisal. Unlike standard diagnostic lab tests, AI outputs are probabilistic suggestions rather than objective facts. Clinicians must be retrained to verify rather than blindly trust these algorithmic recommendations. This is not about teaching clinicians to code but empowering them to be informed and critical users of these new tools.

Finally, the scientific research agenda must shift from a competitive to a collaborative framework. For too long, the focus has been on proving AI can perform a task in isolation, often pitting human against machine. Future research should prioritize understanding and optimizing human-AI collaboration (24). Key questions include how to design intuitive interfaces that support clinical reasoning, how AI tools can be used to strengthen the human therapeutic alliance, and what best practices are for co-designing equitable tools with underserved communities.

In conclusion, the global mental health crisis demands bold solutions, and AI offers tools of undeniable power. The unguided pursuit of automation, however, threatens to replace the genuine human connection at the heart of healing with a fragile simulation. The Augmented Clinician model offers a pragmatic and ethically grounded alternative. It provides a roadmap to harness the power of AI not to simulate humanity, but to empower it. By leveraging technology to support and unburden our human clinicians, we can ensure that as our tools become more intelligent, our care becomes more profoundly human.
